# On Mania

**Published:** 1816-04

**Authors:** Richard Walker


*For the London Medical and Physical Journal.*

### *On Mania;* by Mr. Richard Walker.

MANIA, as is well known, arises from various causes, viz. from a natural organic affection of the brain, which, of course, is incurable by art; and likewise from incidental causes, as injuries, plethora, effusion, &c. which, for the most part, admit of a cure or palliation by art.

Mania arises sometimes from a cause purely mental, and sometimes, as I presume, from metastasis, viz. the translation of a morbid humour from some other part to the head, as in gout, erysipelas, &c. To these alone I mean to confine the present observations.

Mania from metastasis arises ordinarily, I think, from mismanagement either on the part of the professional attendant, the patient himself, or his ordinary attendants; but, in some instances, even under the best management, in consequence of circumstances peculiar to the natural state of the patient, not in every instance to be counteracted.

I by no means intend it should be understood that the translation of a morbid humour, as in gout, erysipelas, &c. from some other part to the head, will necessarily produce a derangement of intellect; but, that it sometimes occurs from this cause, especially when conjoined with other aiding circumstances, I have no doubt, although there be no natural disposition to that affection, in like manner as we occasionally find to be the case from excessive drinking.*

* I am disposed to think, however, that in every instance of mania proceeding from these causes, there is a natural predisposing or remote cause existing in the system, viz. a more than ordinary disposition to excitement, or irritability of mind.

The

The mismanagement to which I allude, on the part of the professional attendant, is by any irregular treatment respecting the administration of internal medicines, improper regimen, or the application to the part itself. With respect to these, I shall briefly observe here, that whoever attempts to check or remove a paroxysm of gout, a severe attack of erysipelas, or, in fact, any humour whatever, especially if generated by the constitutional habit of the person, by any topical means, not consisting in such as might have the power of extracting or carrying off the humour from the habit itself, runs the risk of placing his patient in a very perilous situation, by inducing a translation of such morbific matter to a vital part, viz. the head, chest, &c.

With respect to the management of the patient himself, or his ordinary attendants,—this most commonly consists in a mistaken notion that strong stimulating articles, as brandy, &c. are necessary during a violent inflammatory state of the part from gout, to keep it out of the stomach, as they say; which is not only adding fuel to fire, respecting the complaint itself, but, in the end, by debilitating the habit in general, and the stomach in particular, is the readiest means of producing the effect they intended to obviate; and, moreover, renders the stomach, and the system altogether, less susceptible of the effect of such a stimulus when it may be absolutely required.

Metastasis from gout, unless induced by improper management, occurs more commonly towards the natural termination of a regular fit of gout; that is, when the affection is become torpid, and less fixed to the part affected, and the habit in general debilitated. At which time particular attention is sometimes required, especially in elderly or debilitated persons, to keep it in place till it is quite exhausted or dissipated, and prevent its migrating to other parts—then, and then only, that is, when there is a tendency in the gout to leave the part affected prematurely, is the time for a more cordial as well as invigorating regimen. The regimen and mode of living in general, in gout, must be adapted to the age, constitution, and temperament of the patient: in a young, athletic, or inflammatory patient, there is little danger to be apprehended, but much benefit to be derived, from a moderately low regimen; in a habit the reverse of this, a uniform invigorating regimen, with a due portion of cordials, may be necessary, to keep the gouty affection in its fittest place, carefully avoiding, however, undue excitation.

An extraordinary instance of metastasis, as I take it, but from a different cause, has lately occurred to me. A young gentleman gentleman became my patient for a venereal complaint: the history of the case is briefly this,—he had contracted a venereal complaint about two years before, commencing with chancres, succeeded by buboes in each groin and venereal sore throat; in addition to which, he had a very peculiar affection of the lips, particularly the lower lip, consisting in an exudation of a humour which hardened into a thickish dark-coloured crust, or scab, which, as often as it became dry and separated, was successively renewed.

He had used mercury under the care of a professional gentleman in the country, in the course of which the chancres healed and the buboes burst, but did not heal perfectly; and the lips were no better.

When, in this state, I was first applied to respecting him, my opinion was, as I then stated, that, although there was reason to apprehend there might be some venereal taint remaining, that the affection was not exclusively venereal, but that there was naturally a scorbutic affection in the habit.

I put him under a course of mercurial frictions for about three weeks or a month, at Oxford, by which time the buboes were nearly healed, and his lips somewhat better. Being under the necessity now of retiring to the country, he was directed to pursue the same plan. He came over to me occasionally, appeared to be going on well in every respect excepting the lips, which I had told him I apprehended was not venereal; and that, should this continue after he was in other respects well, I should advise him to try the sea-water. In a short time after, he wrote me word he was perfectly well. I desired him to persist in the application of the medicine some time longer, and to diminish it gradually for some time before entirely relinquishing it.

A fresh tumour, however, appearing in one groin, which came to maturity and burst, and his lips being no better, he became my patient in Oxford on Oct. 27th, 1815. Under the present circumstances, I put him upon a fresh course of mercurial frictions, with the intention of eradicating entirely the venereal virus by a steady cautious perseverance in the specific. In about a fortnight the effect of the mercury became sensible by the breath, gum, &c. and in fact reached an extent beyond what I had intended, viz. a very considerable affection of the mouth, with a copious ptyalism, which compelled me to desist the further use of mercury for the present. In a short time, however, perceiving that the sore of the bubo had receded rather during the interval the mercury was suspended, I resumed it again, when the sore again evidently improved.

About this time he shewed me a tumour on the other groin, groin, of considerable size, hard, not discoloured, and in an indolent state. I directed him now to apply the principal portion (viz. Unguent. Hydrarg. ʒi. o. n.) of the mercury on the thigh of this side, applying a linseed poultice. The tumour still remaining stationary in every respect, I changed this application for the Empl. Ammoniac. cum Hydrarg., renewing it occasionally: this quickly produced a blush on the tumour, with some degree of pain, and it soon burst, discharged properly, and diminished in size; the lips, however, continued nearly in the same state.

Finding, as it now appeared to himself as well as me, that matters were going on as well as could be expected, (the lips excepted, which I had all along apprehended to be inherent in the constitution,) he was directed to continue the use of the mercury, which was so managed, ever since his resuming it, as to keep up only a slight affection of the mouth, which I intended to persist in until the tumour in the groin might heal,—watching, likewise, the effects on the lips.

In a short time he observed to me, that his lips felt better, and that he had no doubt himself (being adverse to my opinion that there was something scorbutic in his habit) that they, as well as his other complaints, would become well by a continued course of the mercury.

In a short time after, I perceived myself that the lips were better, and they became actually well, returning to a perfectly natural state. In a few days after this, however, he exhibited to others evident signs of derangement in the mind, which soon became evident to me, though he continued apparently rational to me after his attendants had perceived him to be otherwise. The derangement of mind increased, accompanied with violent raving;—he became exhausted, and expired.

*Observations on this case.*—It is probable that the following combination of causes concurred in producing the melancholy event: the general debility in the habit, the consequence of a long-continued course of mercury necessary to conquer a venereal affection of long standing, viz. a period of two years, complicated as it was; probably, with a natural constitutional affection. The habit thus reduced, and rendered more irritable, being more susceptible of mental impressions than in health, was, as it appeared, acted upon by some circumstances which deeply affected his mind; and, moreover, as I had reason to believe, the system not kept up by a moderately invigorating regimen, which I had directed, from an idea, on his part, that restraint was essential to his cure. These causes, collectively, might operate in occasioning a translation of the scorbutic humour inherent in the

4 habit, habit, and, as I well know, not radically curable by medicine, to the head; or, it may be possible that the mercury, from the same cause, might, instead of being determined, as usual, to the mouth, or carried off by any other excretory means, exert its influence on the system itself. He was directed, in proportion as the mouth was affected, to drink more or less of diluent liquids.

The mercury of course was desisted from, so soon as this great degree of irritation or mental excitement shewed itself.

The affection, in every respect for which the mercury was applied, was, at the time the mental derangement shewed itself, in as fair a way towards a cure as could be wished, viz. the sore in one groin soundly healed, the other in a fair way, and the lips well; nor had he expressed to me any indisposition in his general health.

The mode of treatment I intended to have adopted, so soon as the derangement of intellect showed itself, would have been, 1st, to have immersed him in the warm bath, placing him afterwards between the blankets, with a view to cleanse his skin of the mercury, and to determine the mercury, if the system were really too strongly impregnated with it, to the surface, and probably to the mouth; 2dly, to have administered opium in small doses, repeating it, and watching its effects, until I might have lulled him to quietness,—which I have repeatedly done in instances of mental derangement, with complete success; and 3dly, conceiving, at this time, that he had lowered himself, contrary to my direction, by spare regimen, &c. endeavour to raise him (for there was evidently a considerable degree of general debility) by a due administration of invigorating diet and cordials, drinking plentifully of warm diluent liquids,*—hoping, by these means, I might, if there was an excess of mercury in the system, bring it externally to the mouth, skin, or other parts; or, if it arose from a translation of a morbid humour of any kind to the head, bring it back, by invigorating the habit, and other auxiliary means, to its former seat, the lips, &c. and in the end restore him, as I have many others, (placed by an apparent metastasis, as in gout, &c. in a very perilous situation, from an atonic cause,) by a similar mode of treatment.

* I at one time suspected that he might not have attended sufficiently to my direction respecting the necessity of supplying the habit, during a course of mercury, with an adequate portion of diluent liquids, a circumstance ever to be attended to; but on enquiry, this apprehension was removed.

It

It is worthy of notice, that at this time, as well as during the whole of the second course of mercury, his mouth was so little affected, that he could masticate and swallow nearly as well as in health, and his appetite not impaired: hence it was almost impossible for me to apprehend he had received an excess of mercury; moreover his breath was to the last so slightly tainted, as to be scarcely perceptible to any one.

In this mode of treatment, however, I was overruled by others of the faculty, who, under the idea that his present affection, perfect mania, requiring the ordinary confinement on such occasions, was, exclusively of other causes, solely the effect of an excess of mercury, and, notwithstanding the evident debility and apparently sinking state of the patient, purged him incessantly to get rid of it, or carry it off, which, of course, tended still further to lower him; when, in a short time, by the effect of this treatment, together with the continuance of almost incessant raving, he became completely exhausted, and expired.

Conceiving this instance of mania to have been of an atonic nature, I presume that any means used with a view to lower the system would have been injurious; and that even topical blood-letting from the temporal artery, the jugular vein, or by leeches, would not have been justifiable.

With respect to the application of counter-stimulants in affections of this nature, such as vesicatories, sinapisms, &c. these enter so regularly into the ordinary routine of practice, that I need not notice them here.

He was under my care ten weeks, in the course of which time he used about six ounces of Unguent. Hydrarg. of the strength only of one-third part of quicksilver.* He took no internal medicine during this course except Epsom salts, or castor oil, occasionally; and, for a certain time, Decoct. Sarsæ Comp. ℔i. per diem.

According to the circumstances detailed, which I have endeavoured to give in a faithful manner, I should apprehend it will be allowed by all experienced unprejudiced practitioners that the remedy, in this instance, was administered in a cautious and justifiable manner; and that the affection, which unexpectedly occurred, and caused a fatal termination, came on in such an insidious manner that scarcely any practitioner might have been aware of it, whatever may have been the circumstances to which it might be attributable.

* The quantity of the unguent used at a time never exceeded a drachm, frequently less; there were likewise occasional interruptions to its use.

attributable. For my own part, I am totally at a loss to conceive how the system can be overcharged with mercury without exhibiting the ordinary evidences of the fact, particularly the mercurial fætor of the breath; nor am I aware that mania might be the probable consequence of an excess of mercury in the habit.

Upon the whole, I think, the mania in this instance, as I have noticed, likewise, in other instances, not the effect of medicine of any kind, was attributable, not to any single circumstance alone, but jointly to a diminished vigour of bodily health, together with an increased irritability of mind, influenced or acted upon by circumstances which, perhaps, in the full vigour of health, and whilst pursuing the ordinary avocations in life, would pass disregarded.* The former, in this instance, the unavoidable consequence of the disease and the necessary mode of treatment, and the latter from incidental circumstances with which I am not exactly acquainted; and the remedy, under these circumstances, that which I had proposed to have adopted, as above mentioned.

In consequence of the unexpected length to which the present part of my subject has extended, I shall defer the remainder to another opportunity.

*Oxford; Feb.* 25, 1816.

P. S.—I observed the Editors' remarks, with the information they contained, annexed to my last paper on Vaccination, with much satisfaction, and wish they might have the effect of reviving the credit of vaccination in such places as it may have sunk in the estimation of the faculty and the public: such is certainly the case at present in Oxford and its vicinity.

Cases of small-pox after vaccination have occurred here since my last communication, to the most experienced and attentive practitioners, which tend still to diminish the credit of that practice, and to extend the practice of inoculation.

---

* A very remarkable instance of this kind having occurred within my knowledge, I shall briefly mention it. A young person, under this predicament, became maniacal, and died, merely in consequence of a well-meant, but ill-timed, zeal in the attempt to produce in this person a reformation from habits of immorality, which was so far overdone as actually to produce the effect mentioned.

